# Mammalian Target of Rapamycin Complex 2 (mTORC2) Is a Critical Determinant of Bladder Cancer Invasion

**DOI:** 10.1371/journal.pone.0081081

**Published:** 2013-11-27

**Authors:** Sounak Gupta, Andrew M. Hau, Jordan R. Beach, Jyoti Harwalker, Elisabetta Mantuano, Steven L. Gonias, Thomas T. Egelhoff, Donna E. Hansel

**Affiliations:** 1 Department of Pathology, University of California San Diego, La Jolla, California, United States of America; 2 Department of Cell Biology, Cleveland Clinic, Cleveland, Ohio, United States of America; 3 Department of Pathology, Cleveland Clinic, Cleveland, Ohio, United States of America; University of Kentucky College of Medicine, United States of America

## Abstract

Bladder cancer is the fourth most common cause of cancer in males in the United States. Invasive behavior is a major determinant of prognosis. In this study, we identified mammalian target of rapamycin complex 2 (mTORC2) as a central regulator of bladder cancer cell migration and invasion. mTORC2 activity was assessed by the extent of phosphorylation of Ser473 in AKT and determined to be approximately 5-fold higher in specimens of invasive human bladder cancer as opposed to non-invasive human bladder cancer. The immortalized malignant bladder cell lines, UMUC-3, J82 and T24 demonstrated higher baseline mTORC2 activity relative to the benign bladder papilloma-derived cell line RT4 and the normal urothelial cell line HU1. The malignant bladder cancer cells also demonstrated increased migration in transwell and denudation assays, increased invasion of matrigel, and increased capacity to invade human bladder specimens. Gene silencing of rictor, a critical component of mTORC2, substantially inhibited bladder cancer cell migration and invasion. This was accompanied by a significant decrease in Rac1 activation and paxillin phosphorylation. These studies identify mTORC2 as a major target for neutralizing bladder cancer invasion.

## Introduction

Bladder cancer accounts for greater than 70,000 new cancer cases in the United States annually, with an estimated global incidence of 386,000 cases per year [1,2] . Urothelial carcinoma (UCa) represents approximately 95% of all bladder cancers, with patient outcomes primarily influenced by pathological grade and stage [3]. Grade is a primary contributor to behavior in non-invasive UCa, which is subdivided into low-grade and high-grade categories. Low-grade disease shows frequent local recurrence on the bladder surface, but rare progression to invasive disease [4]. High-grade disease progresses to invasion in 40-50% of patients [5]. Once invasion occurs, disease-specific survival diminishes with increasing pathological stage (i.e., depth of invasion into the bladder wall), despite therapeutic intervention. 

Bladder cancer remains a relatively understudied disease and the cellular mechanisms that determine cancer progression are incompletely understood. High-grade disease often shows *RB* and *TP53* mutations and *PTEN* deletion; however, these genetic changes can be observed in both invasive and non-invasive lesions. Identification of pro-invasive pathways in bladder cancer has been more challenging. Recent work has shown that fibroblast growth factor receptor-1 (FGFR1) promotes invasion of bladder cancer cells expressing ZEB1, a marker of epithelial-mesenchymal transition (EMT)[6]; FGFR inhibition in this study reduced metastatic spread of orthotopically implanted bladder cancer cells. FOXQ1 expression also was associated with an EMT signature in bladder cancer cells and silencing of FOXQ1 increased E-cadherin expression, decreased vimentin expression and attenuated invasive behavior [7]. The phosphoinositide-3 kinase-AKT-mammalian target of rapamycin (mTOR) pathway has been identified as a candidate pathway in bladder cancer progression in several recent clinicopathological studies [8,9]. Although we have identified a role for mTORC1 in promoting bladder cancer proliferation *in vitro* and bladder cancer xenograft growth *in vivo* [9], the role of mTOR signaling in bladder cancer invasion has not been explored.

mTORC2 is an attractive target for inhibiting bladder cancer invasion because it has been shown to affect cytoskeletal remodeling, cancer cell adhesion, and migration [10-12]. mTORC2 is distinguished from mTORC1 by the presence of the rictor, protor and mSin1 subunits, which are required for full kinase activity [13,14]. Downstream effectors include AKT and the Rho GTPases (Rho, Rac and Cdc42) that regulate adhesion and migration [12,15,16]. mTORC2 also may regulate EMT [17] and has been implicated in invasion and metastasis in colon and breast cancer [10,18]. Selective targeting of mTORC2 reduces cancer metastasis in mouse models [10]; however, its role in bladder cancer invasion and metastasis is unknown.

In this study, we examine the role of mTORC2 in bladder cancer cell invasion. We have used human bladder cancer specimens to show that increased mTORC2 activity is associated with an invasive phenotype. These studies are coupled with *in vitro* and novel *ex vivo* human bladder wall invasion assays to show the critical role of mTORC2 in bladder cancer cell migration and invasion. Alterations in Rho GTPase signaling are detailed. The results from this study indicate that mTORC2 may be an exciting novel target in inhibiting bladder cancer progression.

## Materials and Methods

### Cell culture and reagents

RT4, UMUC3, T24 and J82 cells were purchased from American Type Culture Collection (ATCC, Manassas, VA). HU1 cells were a generous gift from R. Rackley (Cleveland Clinic). Cells were grown in RPMI-1640 (GIBCO, Invitrogen Corp., Grand Island, NY) supplemented with 10% fetal bovine serum (FBS, GIBCO) and antibiotic/antimycotic solution (Sigma, Inc., St. Louis, MO). All cell lines were maintained at 37°C in a humidified atmosphere containing 5% CO_2_. Serum starvation was performed for 16 h by maintaining cells in medium lacking serum, with three phosphate buffered saline (PBS) washes during this time period. To serum-stimulate cells, 10% FBS was added. Rapamycin was obtained from Santa Cruz Biotechnology (Dallas, TX).

### Patient Specimens

All research involving human participants was approved by the Cleveland Clinic IRB and written consent was obtained from patients. Fresh tissue samples were obtained at the time of surgery, flash frozen, and stored at -80°C. Frozen sections were analyzed by microscopy to confirm that greater than >90% of the cells in specimens subjected to immunoblot analysis were tumor cells. Tumor grade was confirmed by a pathologist. For immunoblot analysis, tissue samples were homogenized using the Power Gen 500 homogenizer (Thermo Scientific, San Diego, CA).

### Rictor silencing

siGENOME non-targeting control (NTC) siRNA and siGENOME SMARTpool Rictor-specific siRNA was obtained from Dharmacon (Thermo Scientific). Transfections were performed for 48 h using the Lipofectamine® RNAiMAX Reagent (Invitrogen). Silencing was confirmed by immunoblot analysis.

### Immunoblot analysis

Cells and tissue samples were extracted in RIPA buffer containing protease inhibitor cocktail (Sigma) and phosphatase inhibitor cocktails 1 and 2 (Sigma) and subjected to SDS-PAGE on 4-15% gradient gels. Proteins were transferred to Hybond ECL membranes (GE Healthcare, Pittsburgh, PA) using the Bio-Rad Transblot semidry transfer system (Life Science Research, Hercules, CA). Membranes were blocked with 1% bovine serum albumin and incubated with primary antibodies overnight at 4°C in blocking solution. Antibodies, which were obtained from Cell Signaling Technology (Danvers, MA), targeted rictor (1:1000), p-AKT (Thr308; 1:1000), p-AKT (Ser473; 1:1000), pan-AKT (1:2000), p-S6 (1:2000), total-S6 (1:2000), and actin (1:5000). Blots were incubated with alkaline phosphatase-conjugated secondary antibodies (Jackson ImmunoResearch, West Grove, PA) at room temperature for 1 h and developed using ECF™ Western blotting reagent packs (GE Healthcare). Densitometry was performed using Imagequant software (GE Healthcare).

### Cell spreading and morphometric analysis

Cells were trypsinized and plated at low density on borosilicate chamber coverglass slips (Nalge Nunc International, Naperville, IL) coated with 20 µg/ml fibronectin. Time lapse imaging was performed using a Leica TCS-SP2 with a Plan-Apochromat 63x/1.4 N.A. oil objective (Leica, Buffalo Grove, IL) for 24 h and cell morphology was analyzed using Image J software (http://rsbweb.nih.gov/ij/). The total area of individual cells was quantified for each corresponding time point and plotted with respect to elapsed time.

### Modified scratch wound migration assays

Modified scratch wound migration assays were performed as previously described [19]. Briefly, tissue culture plates were pre-treated with 20 ng/mL fibronectin in PBS for 1 h. A thin strip of polydimethylsiloxane (PDMS) was placed in the middle of each well and allowed to adhere. Cells were seeded on top and incubated for 18 h, after which the PDMS strips were removed to reveal an unperturbed fibronectin matrix. Multiple fields of view per well were imaged at regular intervals using the Leica DM IRE2 microscope (Leica) and MetaMorph imaging software (Molecular Devices, Sunnyvale, CA).

### Transwell invasion assays

Transwell invasion assays were performed as previously described [9] using 8 micron PET membranes (Corning Life Sciences, Tewksbury, MA) coated with Matrigel (BD Biosciences, San Jose, CA). Cells were seeded onto membranes in serum-free medium (SFM) and allowed to migrate for 24 h towards a paired chamber containing medium supplemented with 10% FBS. Membranes were fixed with 4% paraformaldehyde, permeabilized in 0.1% Triton X-100, and stained with DAPI. The top of the membrane was wiped clean with a cotton swab so that only transmigrating cells were counted. Fields of view at 200x magnification were randomly imaged using the Leica DMR microscope and the number of cells present was quantified using ImageJ software.

### Bladder wall invasion assay

For bladder slice assays, fresh, full-thickness slices of the bladder wall were obtained from patients undergoing radical cystectomy and were obtained from disease-free portions of the bladder wall by a urologic pathologist. The basement membrane was stripped to expose the connective tissue (lamina propria) and the external aspect of the perivesical fat was embedded in agar. Tissue was maintained in RPMI/10% FBS with antibiotics/antimycotics for 7-10 days. Bladder cancer cells were labeled with CellTrackerTM Red, seeded onto the tissue slice, and incubated for 3-7 days with regular changes of medium at 24 h intervals. In order to assess invasive depth of tumor cells, tissue was perpendicularly sectioned at 10 micron intervals to see all tissue layers and cells were visualized using hematoxylin and eosin (H&E) staining, immunohistochemistry and immunofluorescence. Images were captured using a Leica DMR fluorescent microscope and Spot Advanced software (SPOT Imaging Solutions, Sterling Heights, MI). Depth of invasion was measured using Image-Pro Plus software on captured images (Media Cybernetics, Rockville, MD). Immunohistochemistry to detect cytokeratin AE1/3 was performed as previously described [9] using a Discovery XT automated stainer. 

### Immunofluorescence microscopy

Immunofluorescent staining was performed as previously described [20]. Cells were fixed in PBS supplemented with 2 mM MgCl_2_, 2 mM EGTA, and 4% formaldehyde, permeabilized in PBS containing 0.5% Triton X-100, incubated with p-paxillin antibody (Cell Signaling; 1:1000) and visualized with anti-rabbit Alexa-conjugated secondary antibodies (Invitrogen). Nuclei were stained using DAPI.

### Rac1/RhoA activation assay

RhoA/Rac1 activation assays were performed as described by the manufacturer (Cell Biolabs, Inc., San Diego, CA). Briefly, cells were grown to 50-70% confluency, treated with FBS for 30 min and transfected with siRNA 72 h prior to harvesting. Control samples were incubated with GTPγS. Equivalent amounts of protein were incubated with either PAK1-PBD agarose or Rhotekin-PBD agarose (Millipore, Billerica, MA) for 1 h at 4°C. The agarose-conjugated beads were washed 3 times in PBS and boiled in SDS–PAGE sample buffer. Immunoblotting was performed with primary antibodies that detect Rac1 (BD Biosciences; 1:1000) and RhoA (Cell Signaling; 1:1000).

## Results

### mTORC2 signaling is increased in invasive human bladder cancers

**Figure 1:**
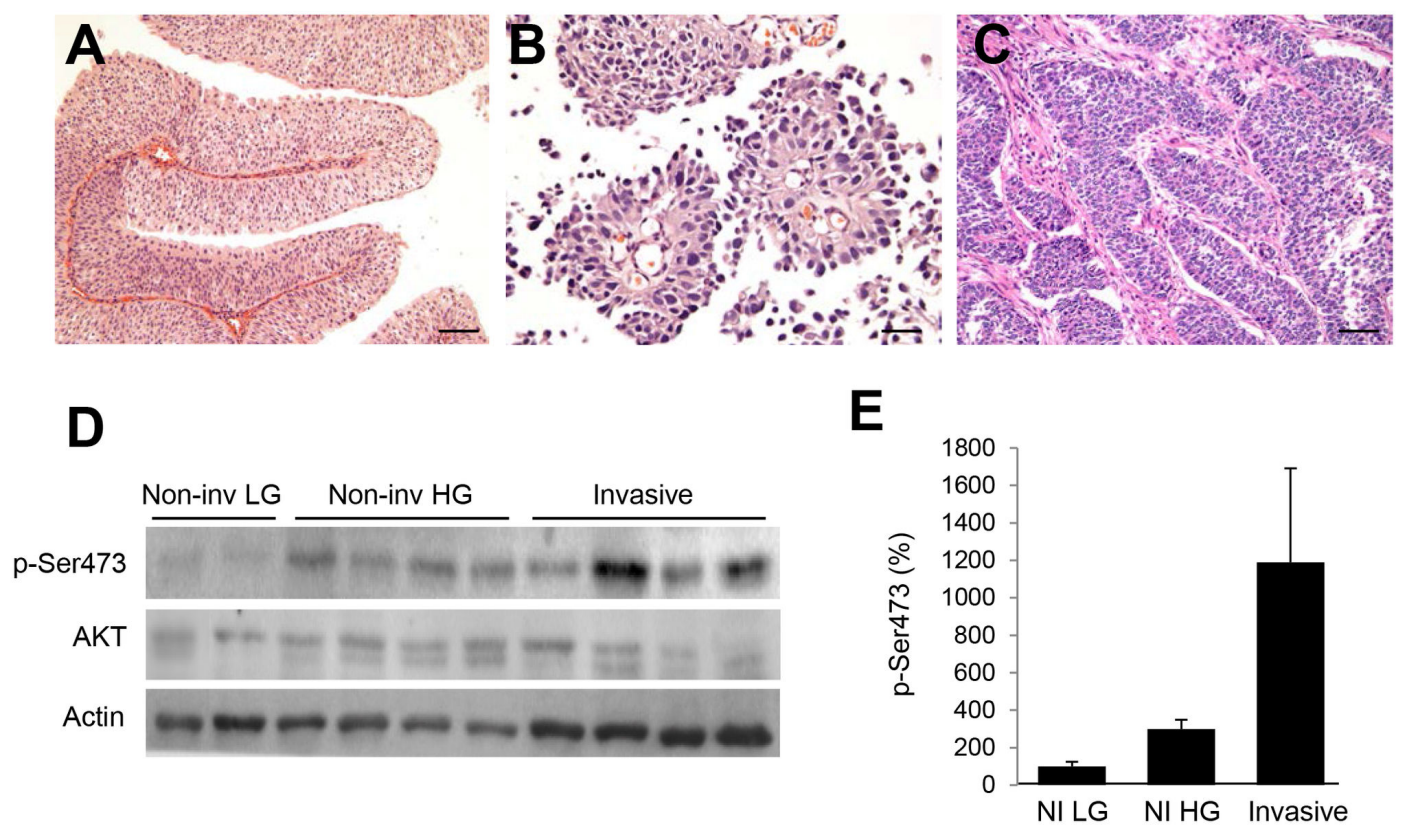
Increased mTORC2 activity occurs in invasive bladder cancer. We compared *A*, non-invasive low-grade papillary UCa (non-inv LG; scale bar 80 microns); *B*, non-invasive high-grade papillary UCa (non-inv HG; scale bar 80 microns); and *C*, invasive high-grade UCa to determine mTORC2 activity (scale bar 100 microns). *D*, immunoblotting for p-Ser473 as a marker of mTORC2 activity was performed and shows higher mTORC2 activity in both non-invasive HG and invasive lesions. *E*, densitometry was used to quantify p-Ser473/ total AKT signal intensities; averages and standard error of the mean (SEM) were normalized to the average signal intensity for the non-invasive LG samples.

Non-invasive low-grade (LG) UCa is defined by papillary fronds lined by urothelium with retained polarity and only minimal cytologic atypia (Figure **1A**). This histology correlates with frequent mutations in *FGFR3* and *RAS* [21]. In contrast, non-invasive high-grade (HG) UCa shows nuclear enlargement, hyperchromasia, and loss of polarity that is commonly associated with mutations in *TP53* and *RB* genes (Figure **1B**). In a subset of HG UCa cases, invasive behavior is observed (Figure **1C**) and this is associated with worsened outcomes. We evaluated mTORC2 activity in samples of LG UCa, HG UCa, and HG UCa with invasion by determining phosphorylation of AKT at Ser473 (p-Ser473). Immunoblotting suggested an increase in p-Ser473 in non-invasive HG UCa compared with LG lesions (Figure **1D**) and an additional increase in p-Ser473 in invasive HG lesions. Densitometry comparing p-Ser473/total AKT ratios was performed, with results normalized to non-invasive LG UCa (Figure **1E**). Although the small number of samples precluded statistical significance, p-Ser473 appeared increased in the more advanced pathologies.

### mTORC1 and mTORC2 Are Activated in Malignant Bladder Cancer Cells

**Figure 2:**
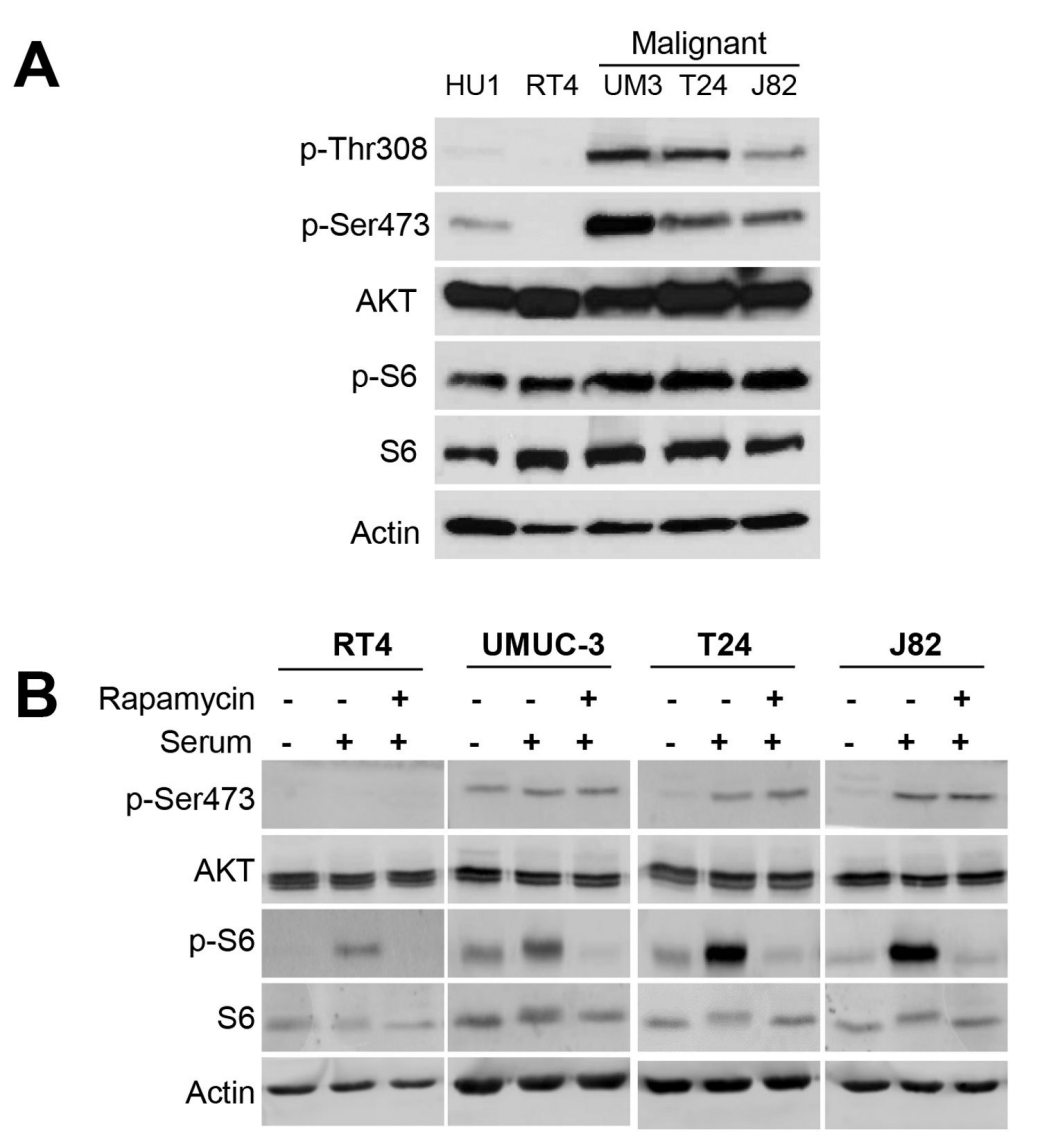
Increased mTORC1 and mTORC2 activity is observed in malignant bladder cancer cells and can be induced with serum. *A*, immunoblotting shows higher mTORC1 (p-S6) and mTORC2 (p-Ser473) activity in malignant UMUC-3, T24 and J82 bladder cancer cells, compared with normal urothelial HU1 cells and benign RT4 cells. *B*, serum stimulation induced mTORC2 activity in malignant bladder cancer cells. mTORC1 signaling was responsive to rapamycin in this model; mTORC2 was not.

We compared mTORC1 and mTORC2 signaling in hTERT-immortalized normal urothelial cells (HU1) [22], benign bladder papilloma-derived RT4 cells and in invasive UMUC-3, T24, and J82 bladder cancer cells. mTORC1 activity was determined by measuring phosphorylation of p70 S6 kinase (p-S6), whereas mTORC2 activity was determined by p-Ser473 AKT levels. Under baseline conditions, in serum-containing medium, HU1 and RT4 cells showed low levels of AKT phosphorylation at threonine 308 (p-Thr308), which reflects activation of PI3K and PDK1 upstream of AKT (Figure **2A**). p-Ser473 and p-S6 also were low in HU1 and RT4 cells. The three malignant bladder cancer cell lines demonstrated increased p-Thr308, p-S6, and p-Ser473 levels.

Next we evaluated mTORC1 and mTORC2 activity in bladder cancer cells that were made quiescent by serum starvation and then stimulated by adding serum (Figure **2B**). In RT4 cells, p-Ser473 was barely detectable before and after serum stimulation. In the T24 and J82 cells, p-Ser473 was minimal after serum starvation but increased substantially upon addition of serum. UMUC-3 cells demonstrated p-Ser473 even after serum starvation and a modest increase in p-Ser473 after adding serum. These results suggest that mTORC2 is activated in response to serum stimulation in invasive bladder cancer cells. In contrast, p-S6 increased in response to serum stimulation in benign as well as malignant bladder cancer cells. Low dose rapamycin (10 nM), an inhibitor of mTORC1, selectively blocked p-S6 and had little or no effect on mTORC2 activity [9].

**Figure 3:**
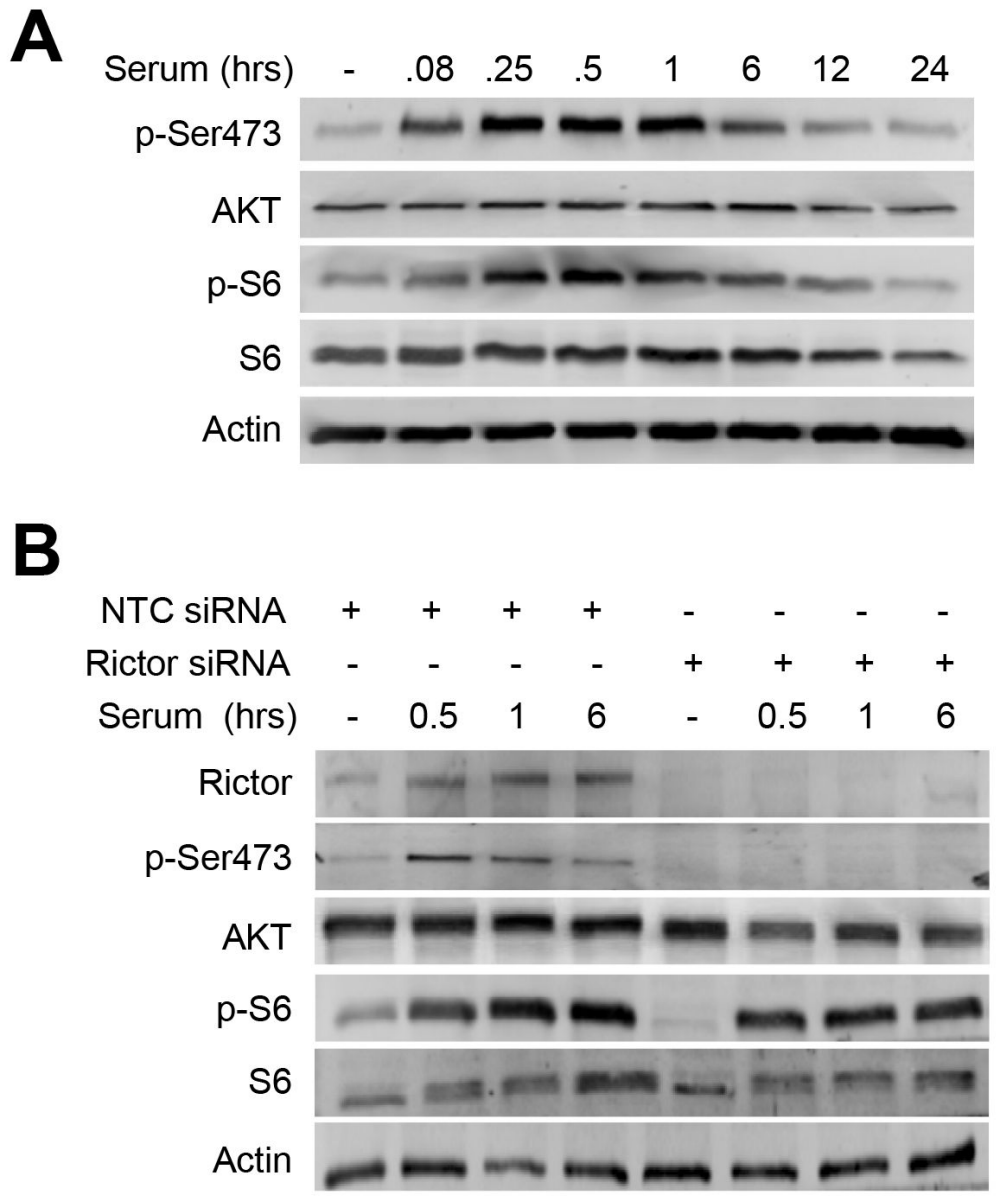
Temporal profiling of mTORC2 activity in malignant bladder cancer cells shows an early response to serum stimulation. *A*, serum stimulation in J82 cells shows high levels of mTORC2 (p-Ser473) activity by 5 min that persists through 6 h. *B*, rictor gene-silencing using pooled siRNA dramatically reduces detectable rictor protein and ablates p-Ser473 relative to that observed in cells transfected with NTC siRNA. No effect on mTORC1 signaling (p-S6) was detected.

In time course experiments in J82 cells, mTORC2 activity increased rapidly following serum stimulation and was sustained through at least 6 h in culture (Figure **3A**). The kinetics of mTORC2 (p-Ser473) activation and deactivation were similar to those identified for mTORC1 (p-S6). To confirm that p-Ser473 specifically reflected mTORC2 activity, we silenced rictor in J82 cells using pooled siRNA. As shown in Figure **3B**, rictor gene-silencing was effective and completely ablated phosphorylation of the Ser473 residue on AKT in response to serum. Phosphorylation of the mTORC1 target, S6, was unaffected, demonstrating specificity in the silencing event. Non-targeting control (NTC) siRNA did not alter rictor levels or response to serum stimulation. Similar results were obtained in experiments with T24 cells (results not shown). These findings demonstrate effective rictor gene-silencing and an associated reduction in mTORC2 signaling.

### mTORC2 is a critical regulator of cell migration and invasion in bladder cancer

**Figure 4:**
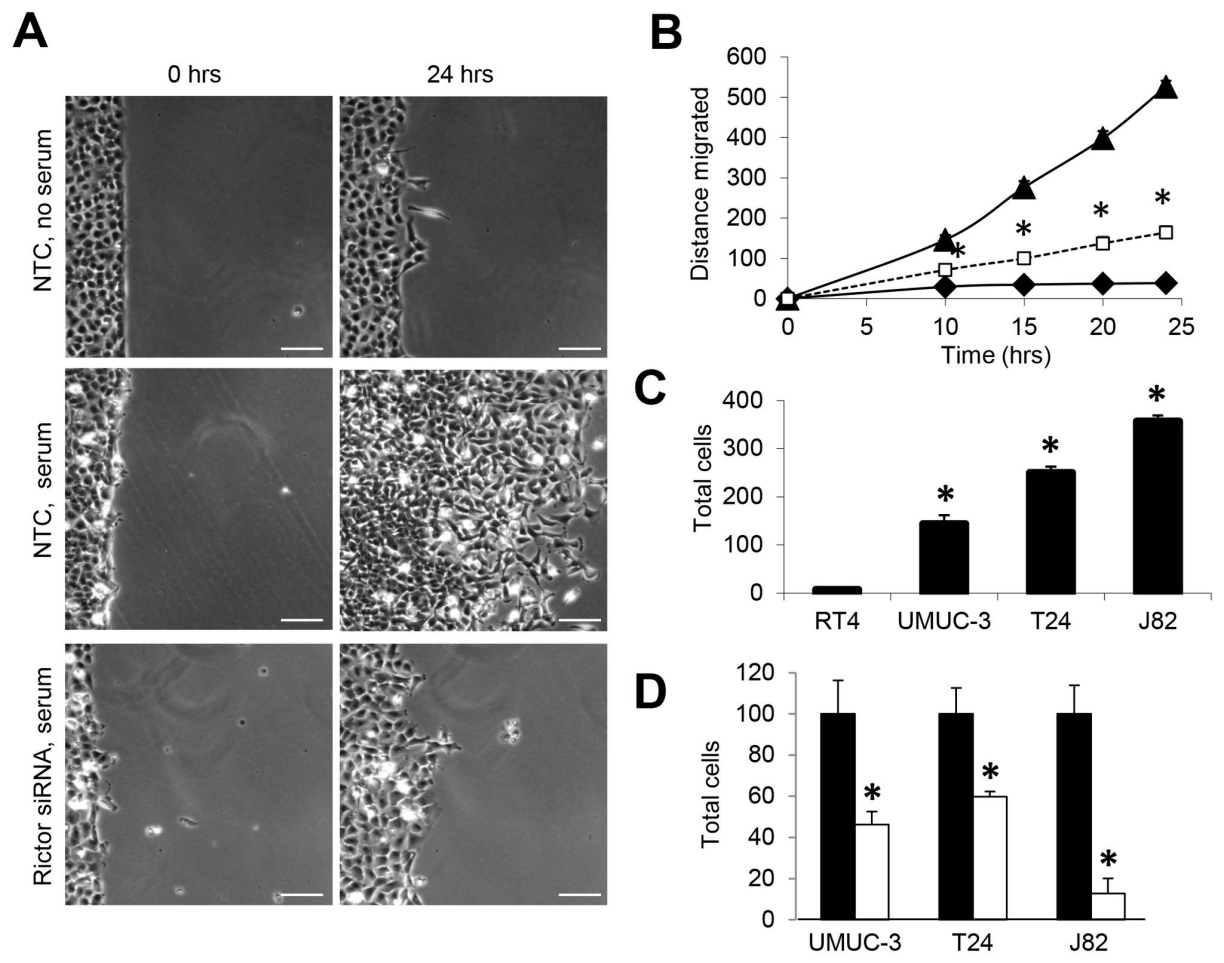
mTORC2 is critical in bladder cancer cell migration and transwell invasion. *A*, serum starvation blocked J82 bladder cancer cell motility. Addition of serum induced robust migration of J82 cells that were transfected with NTC siRNA, as determined using a modified scratch assay. Migration was inhibited by rictor gene-silencing. Scale bar 100 microns. *B*, quantification shows a reduction in the distance of migration of cells transfected with rictor-specific siRNA (□) compared with cells transfected with NTC siRNA (▲). Also shown are cells that were no serum-stimulated (♦). A total of 6 frames were analyzed for each time point per condition. *C*, transwell invasion on Matrigel-coated membranes shows higher numbers of malignant bladder cancer cells that invade. D, rictor gene-silencing significantly reduces cell invasion of all three malignant bladder cell lines in transwell assay. (*) statistically significant comparison using student t-test, p<0.05).

We used a modified scratch-wound assay to assess cell migration in T24 bladder cancer cells that were transfected with NTC or rictor-specific siRNA (Figure **4A**). The substratum was fibronectin-coated. In cells transfected with NTC siRNA, substantial migration was observed only in the presence of serum. Rictor-specific siRNA reversed the effects of serum on cell migration. Figure **4B** summarizes the effects of rictor-silencing on T24 cell migration as a function of time. Statistically significant decreases in cell migration were associated with rictor gene-silencing from 10 to 24 h. Similar results were obtained when rictor was silenced in J82 cells and cell migration was studied (results not shown).

Bladder cancer cell invasion was initially studied using reconstituted Matrigel in Boyden chambers. Each of the three malignant bladder cancer cell lines demonstrated significantly increased invasion through Matrigel, compared with the non-malignant RT4 cells (Figure **4C**). Rictor gene-silencing significantly decreased invasion by the malignant bladder cancer cells (Figure **4D**). In J82 cells, invasion was inhibited by greater than 80%. The efficacy of rictor gene-silencing suggested that mTORC2 may be a major target for inhibiting invasion of bladder cancer.

### Human bladder wall invasion by bladder cancer cells requires mTORC2

**Figure 5:**
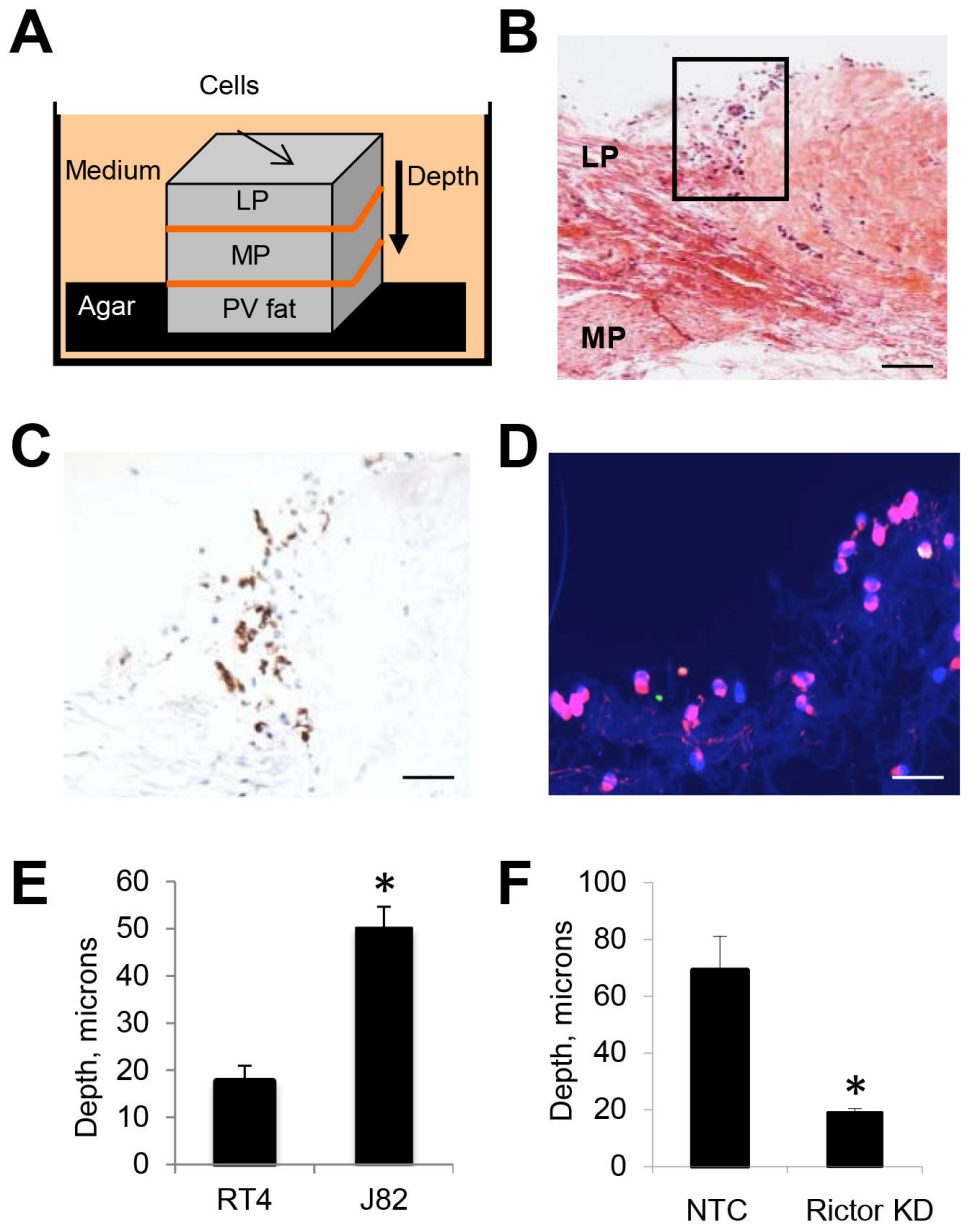
mTORC2 regulates invasion of bladder cancer cells into the human bladder wall. *A*, schematic showing orientation of human bladder wall invasion assay that includes lamina propria (LP), muscularis propria (MP) and perivesical fat (PV fat). B, H&E section showing invading J82 cells in the lamina propria after 72 h (scale bar 200 microns) *C*, cells highlighted in previous panel showed positive immunostaining for pancytokeratin (scale bar 100 microns). *D*, immunofluorescent stain shows invasion of malignant J82 cells (red), at 72 hr (scale bar 50 microns). *E*, quantification of tissue invasion by RT4 and J82 cells (microns). *F*, rictor gene-silencing significantly inhibits invasion of bladder all by J82 cells. (*) statistically significant comparison using student t-test, p<0.05).

To further support our hypothesis that mTORC2 may be critical in regulating bladder cancer cell invasion, we developed a novel *ex vivo* model system to test invasion of the normal bladder wall. Full-thickness sections of adult human bladder that included lamina propria (LP; fibroblast-predominant connective tissue with blood vessels), muscularis propria (MP; dense bundles of smooth muscle) and peri-vesical fat (PV fat; primarily fibroadipose tissue and capillaries) were anchored in agar and bathed in cell culture medium (Figure **5A**). We seeded these preparations with fluorescently-labeled malignant J82 cells and allowed these cells to penetrate the bladder wall for 72 h. J82 cells that were subjected to rictor gene-silencing or treated with NTC siRNA also were inoculated on the bladder wall preparations. Invading cells were readily identified by light microscopy (Figure **5B**). Immunohistochemical staining for pancytokeratin (cytokeratin AE1/3) confirmed that the invading cells were epithelial in origin (Figure **5C**). Fluorescent imaging of invading cells is shown Figure **5D**. A significant increase in invasion was observed when the malignant J82 cells were compared with RT4 cells (p<0.05; Figure **5E**). Rictor gene-silencing in the J82 cells significantly reduced invasive depth (Figure **5F**), indicating an essential role for mTORC2 in regulating J82 cell invasion in our *ex vivo* human model system.

### Rac1 is a downstream effector of mTORC2 in bladder cancer cells

**Figure 6:**
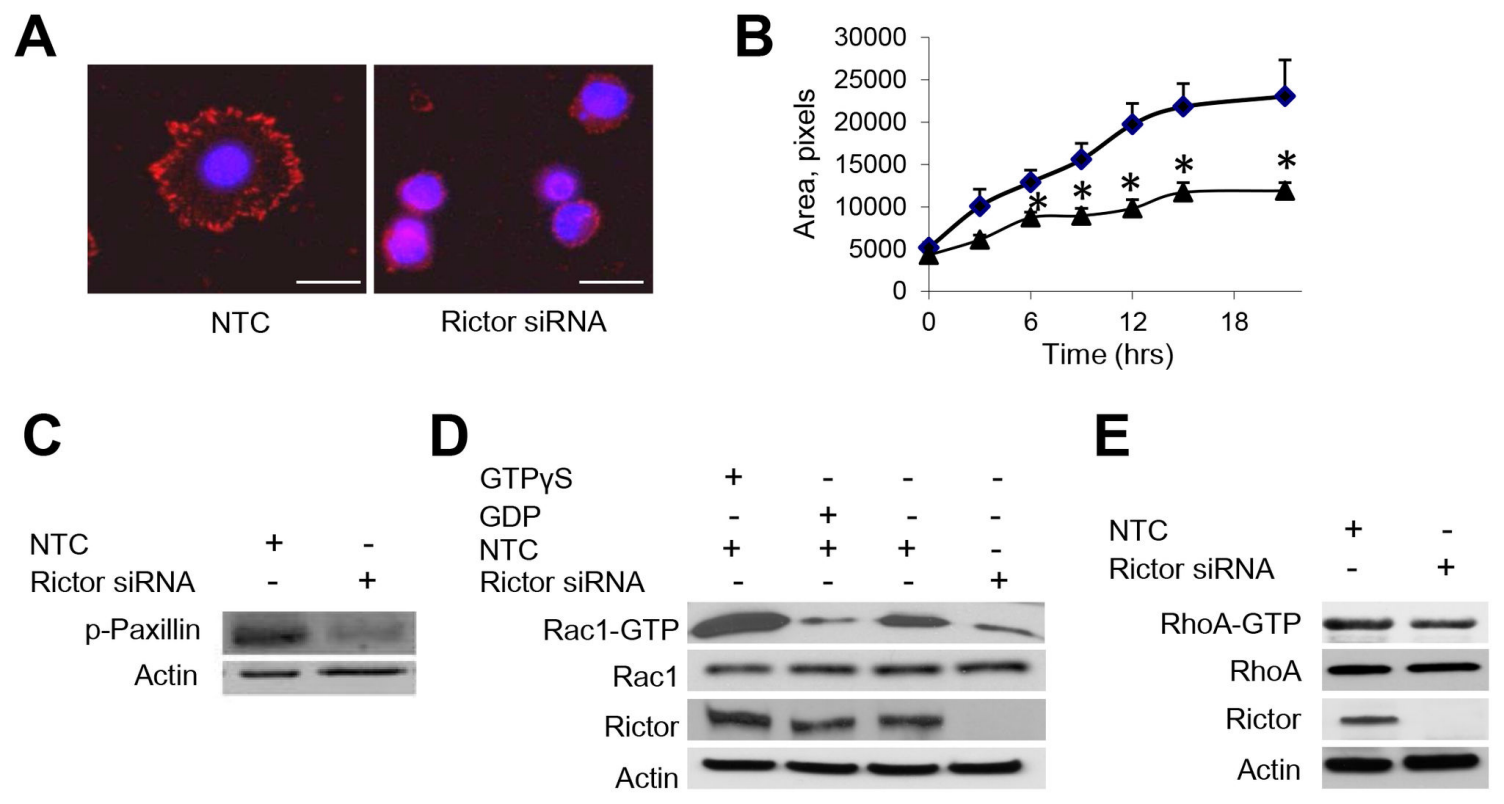
mTORC2 regulates cell spreading and Rac1 activity in bladder cancer cells. J82 cells were either transfected with rictor-specific siRNA or NTC siRNA 72 h prior to the start of the experiment. *A*, immunofluorescent staining shows broad cytoplasmic processes and peripheral phospho-paxillin in cells that are transfected with NTC siRNA. This is reduced in cells with rictor gene-silencing. Scale bar 30 microns. *B*, cell-surface areas were determined for J82 cells transfected with NTC (♦) or rictor-specific siRNA (▲) and allowed to spread for the indicated times (mean±SEM, n>15 for each time point; (*) p<0.05). *C*, immunoblotting for phospho-paxillin in J82 cells that were transfected with rictor-specific or NTC siRNA and allowed to adhere for 30 min. *D*, GTP-Rac1 was determined in J82 cells transfected with NTC or Rictor-specific siRNA. Rictor knockdown reduced Rac1 levels to 60.2% of control. Cells with GTPγS were analyzed as a positive control and GDP as negative control. *E*, GTP-loaded RhoA in J82 cells transfected with rictor-specific or NTC siRNA. Rictor knockdown reduced RhoA levels to 83.3% of control.

Next, we examined the effects of rictor gene-silencing on J82 cell spreading. Cells that were transfected with NTC siRNA and allowed to spread on fibronectin showed broad cytoplasmic extensions and a flattened morphologic appearance (Figure **6A**). In contrast, rictor gene-silencing yielded smaller ovoid cells that failed to spread. Quantification of the surface area covered by adherent cells confirmed that rictor gene-silencing significantly attenuated spreading of J82 cells (Figure **6B**). We next used J82 cells to evaluate phosphorylation of paxillin, which regulates focal adhesion formation and turnover and may regulate lamellipodium formation[23]. Rictor gene-silencing reduced the level phospho-paxillin in malignant J82 cells relative to NTC transfected cells. Analysis of Rac1 activation in J82 cells showed a substantial reduction in the level of GTP-loaded (activated) Rac1 (Rac1-GTP) when rictor was silenced (Figure **6D**). Densitometry showed that rictor knockdown reduced Rac1 levels to 60.2% of rictor-expressing cells. By contrast, GTP-activated RhoA was modestly decreased by rictor silencing, with rictor knockdown reducing RhoA activity to 83.3% of rictor-expressing cells (Figure **6E**). 

## Discussion

In bladder cancer, local invasion into the muscularis propria (detrusor muscle) is an important determinant of prognosis and subsequent patient therapy. Signaling pathways that are uniquely involved in bladder cancer cell invasion should be prime targets for therapeutics development. However, commonly described genetic and signaling alterations in bladder cancer have not been considered in the context of invasion. HG UCa, which develops invasive behavior in a subset of patients, often harbors mutations in *TP53* and *RB*. Mutations and/or loss of these oncogenes have been implicated in defective cell cycle regulation [21]. Alterations in phosphatase and tensin homolog (PTEN) lipid/protein phosphatase have been demonstrated in approximately 30% of patients with bladder cancer and encompass homozygous gene deletion, loss of heterozygosity (LOH), and gene mutation [24]. However, the ultimate impact of *PTEN* on downstream signaling cascades such as mTORC2 in bladder cancer invasion is unclear. 

In this study, we demonstrated that mTORC2 activity correlates with human bladder cancer grade and invasion. At the mechanistic level, mTORC2 controls bladder cancer cell spreading, migration, and invasion of Matrigel. mTORC2 also regulates bladder cancer invasion in a novel *ex vivo* human bladder model that captures the complex microenvironment of the bladder wall. Increased mTORC2 activity results in activation of Rac1, which is known to promote cell spreading and migration [25]. These results suggest that activated mTORC2 may constitute a critical and targetable signaling element in bladder cancer invasion.

mTORC2 represents one of two signaling arms of the mTOR cascade. mTORC2 exists as a complex that includes mTOR and mLST8 and is distinguished from the mTORC1 by the presence of rictor, protor and mSIN1 [26]. In contrast with the mTORC1 complex that regulates translation in response to nutrient status and growth factors, mTORC2 controls processes that require cytoskeletal re-modeling, such as cell spreading and migration. mTORC2 may be activated by insulin-stimulated PI3K, which promotes association of mTORC2 with ribosomes [27,28]. Cyclic adenosine monophosphate (cAMP) and glycogen synthase kinase-3β inhibit mTORC2 activation [16,29]. 

Once activated, mTORC2 phosphorylates Ser473 in AKT, which has been proposed to regulate AKT activity and stability[30]. Rho family GTPases are reported to function as major downstream effectors of mTORC2 [10,12,17,31]. Neutrophil migration towards chemoattractants is regulated by mTORC2 by a mechanism that involves MyoII and regulation of RhoA activity [32]. In colon cancer cells, rictor gene-silencing induces mesenchymal-epithelial transition (MET) and inhibits cell motility by effects on both RhoA and Rac1[10]. Whereas reduced E-cadherin has been reported in the malignant cell lines UMUC-3, J82 and T24 cell lines relative to RT4 cells [6], knockdown of rictor in our model system does not appear to reverse this phenotype (data not shown). 

In our experiments with J82 bladder cancer cells, rictor gene-silencing substantially decreased Rac1 activation while having a more modest effect on RhoA. In many cell systems, pathways exist to regulate Rac1 and RhoA reciprocally[33]. Thus, even a modest decrease in RhoA activation in association with rictor gene-silencing may be significant in the bladder cancer cells. The marked effects of rictor silencing on cell spreading, migration, and invasion are consistent with our biochemical results suggesting that Rac1 is major target of mTORC2 in bladder cancer. One caveat is that rictor downdown also reduces p-Ser473, which may induce additional downstream changes in AKT signaling that could impact invasion independently of Rho GTPase activity.

When comparing mTORC2 activation in bladder cancer cell lines, we found that UMUC-3 cells were unique in that detectable p-Ser473 was present even in the absence of serum. The increase in the basal level of p-Ser473 in UMUC-3 cells most likely reflects the homozygous null deletion of *PTEN* in these cells [9,34], eliminating an important suppressor of mTOR signaling. UMUC-3 cells may represent a model for bladder cancer cells in which *PTEN* is mutated. Cancers with *PTEN* deletion or mutation are frequently resistant to targeted anticancer agents that function upstream of PTEN such as those against epidermal growth factor receptor[35]. The T24 and J82 cells may be preferred models for bladder cancer that occurs in the absence of *PTEN* mutation. These cells demonstrated essentially undetectable levels of P-Ser473 in the absence of serum and strong stimulation of mTORC2 activity when serum was added. 

The involvement of Rac1 as a major downstream target of mTORC2 in the regulation of bladder cancer invasion may link a number of recent findings in the literature. One study showed that Rac1 regulates bladder cancer cell invasion and actin reorganization downstream of integrin-linked kinase (ILK) [36]. Another group has reported that rictor can physically interact with ILK [37] to induce phosphorylation of AKT at Ser473, which represents an mTORC2-specific signature. Rac1 has been reported to directly interact with mTOR in a GTP-independent manner [38]. 

In conclusion, our studies demonstrate that mTORC2 is a critical regulator of bladder cancer migration and invasion, with effects mediated primarily through Rac1. Selective targeting of mTORC2 in invasive bladder cancer may represent a novel approach to patient therapy to prevent or limit invasion of bladder cancer. 
